# Impact of Oral Intake of Glucosylceramide Extracted from Pineapple on Xerostomia: A Double-Blind Randomized Cross-Over Trial

**DOI:** 10.3390/nu11092020

**Published:** 2019-08-28

**Authors:** Mamoru Murakami, Yasuhiro Nishi, Kae Harada, Tomohiro Masuzaki, Yoko Minemoto, Takahiro Yanagisawa, Takaharu Shimizu, Akito Tsuboi, Taizo Hamada, Masahiro Nishimura

**Affiliations:** 1 Denture Prosthodontics Restoration, Advanced Dentistry Center, Kagoshima University Hospital, 8-35-1 Sakuragaoka, Kagoshima 890-8544, Japan; 2 Departments of Oral and Maxillofacial Prosthodontics, Field of Oral and Maxillofacial Rehabilitation, Advanced Therapeutic Course, Kagoshima University Graduate School of Medical and Dental Sciences, 8-35-1 Sakuragaoka, Kagoshima 890-8544, Japan; 3 Division of Community Oral Health Science, Department of Community Medical Supports, Tohoku Medical Megabank Organization, Tohoku University, 2-1 Seiryo-machi, Aoba-ku, Sendai 980-8573, Japan; 4 Hiroshima University, Kasumi 1-2-3, Minamiku, Hiroshima 734-8553, Japan

**Keywords:** dry mouth, xerostomia, glucosylceramide, pineapple

## Abstract

*Background:* The aim of this double-blind randomized cross-over trial was to evaluate the effect of oral intake of glucosylceramide extracted from pineapple on oral moisture and xerostomia symptoms. *Methods:* Sixteen participants who had xerostomia symptoms were randomly allocated into two groups. One group received, as test samples, tablets containing glucosylceramide extracted from pineapple (GCP) followed by placebo tablets. The other group received the test samples in the reverse order. Participants were instructed to take tablets of the first test sample once a day (after breakfast) for two consecutive weeks. Then, after a washout period of four weeks, participants were instructed to take the other test sample for two consecutive weeks. The oral moisture level of the lingual mucosa, xerostomia symptoms, and the number of fungiform papillae was evaluated. *Results:* The oral moisture significantly increased, and the visual analog scale (VAS) of “How is the dryness of your mouth?” significantly improved after GCP tablets intake and not after placebo tablets intake. The number of fungiform papillae was not significantly different following the intake of GCP tablets or placebo tablets. *Conclusion:* Results suggested that oral intake of GCP may improve the moisture level and xerostomia symptoms.

## 1. Introduction

Xerostomia can be caused by radiation therapy, Sjogren’s syndrome, and multidrug therapy in older patients [1,2]. Xerostomia can increase the risk of opportunistic infections, dental caries, periodontal disease, decreased denture retention, and traumatic ulcers, resulting in a negative effect on quality of life [1,2].

For xerostomia resulting from radiotherapy or Sjogren’s syndrome, pilocarpine and cevimeline are prescribed to promote saliva secretion [1,2]. However, these drugs can cause adverse side effects such as hyperhidrosis and dyspepsia [1,2]. Therefore, as a symptomatic treatment for xerostomia, oral moisturizers can be prescribed [1,2,3]. However, these products still need further improvement in terms of the prolonged duration of treatment effect and cost effectiveness [2,3].

Ceramide is a major component in lipid layers between stratum corneum cells [4] and plays an important role in the living body; specifically, it has a barrier function against harmful substances from the environment and moisture retention function to prevent water loss [5]. However, ceramide decreases due to various factors including aging, stress, and seasonal changes, and the decrease in ceramide is strongly associated with pathological conditions of the skin, including dry skin [5,6,7,8]. Glucosylceramide is a glucoside of ceramide, and is a major sphingolipid in plants such as pineapple, wheat, rice, corn, soybean, and konjac [9]. Orally ingested glucosylceramide gets hydrolyzed to become a sphingoid base that is absorbed in the intestine [10], and it enhances the synthesis of ceramide [11]. In Japan, glucosylceramide extracted and purified from pineapple fruits (GCP) has been reported to be safe for consumption and has been approved as food [12,13].

It has been reported that the oral administration of GCP can improve the barrier function and tone of the skin by promoting the synthesis of ceramide and the turnover of skin cells [11,14,15]. In the same manner as the skin, the lingual mucosa consisting of the stratum corneum squamous epithelium may lead to atrophic tongue due to a dry condition in the oral cavity [16]. In principle, if the oral intake of GCP provides beneficial effects including ceramide synthesis and turnover of cells in lingual mucosa, it would be expected that oral moisture level would increase, leading in turn to an improvement of xerostomia condition. Such a treatment for xerostomia might be a natural alternative to industrial products and chemicals such as oral moisturizers. However, to date, there has been no quantitative investigation of possible improvements of xerostomia by the oral administration of GCP.

The purpose of this study was to measure, by means of a double-blind randomized cross-over trial, the beneficial effects of oral intake of GCP on oral moisture and xerostomia symptoms.

## 2. Materials and Methods

### 2.1. Study Design

This prospective, double-blind, randomized controlled, cross-over trial was conducted at a single center in Japan. The study protocol was reviewed and approved by the Ethical Committee of Kagoshima University Hospital (#27-239) and followed the guidelines of the amended Declaration of Helsinki. This study was registered with the University Hospital Medical Information Network (UMIN ID: 000022976).

**Figure 1:**
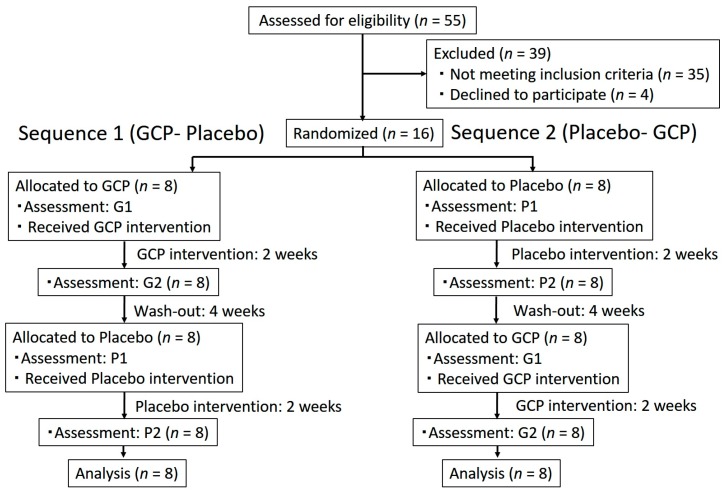
G1: Moisture level measured before taking GCP tablets, G2: Moisture level measured after taking GCP tablets, P1: Moisture level measured before taking Placebo tablets, P2: Moisture level measured after taking Placebo tablets. Flow chart for our randomized crossover trial. (G1 and G2) and (P1 and P2) are before and after taking the glucosylceramide extracted from pineapple (GCP) and placebo test sample, respectively.

Participants were randomly allocated into two groups; namely, Sequence 1, a group that took GCP tablets first and placebo tablets second (GCP-Placebo); and Sequence 2, a group that took tablets in the reverse order (Placebo–GCP) (Figure 1). In this study, participants and researchers were double blinded. Before registration of the participants, a researcher (Y.M.) who was not informed of the research protocol created randomized codes (1:1 ratio, four-block size) by using Microsoft Excel 2013 (Microsoft) to avoid allocation bias. Two researchers (K.H., T.M.) registered participants, and an assignor (T.Y.) who was not involved in measurements assigned each participant to Sequence 1 or 2. Then, the first blinded test sample was provided by the assignor to the participants.

Participants were instructed to take a tablet of the test sample once a day, after breakfast, for two consecutive weeks. After taking the tablet for two weeks, four weeks of a washout period followed. Then, at the end of the washout period, participants received the next blinded test sample from the assignor, and were instructed to take it for two weeks in the same manner as the first one. A researcher (M.M.) who was not involved in the allocation conducted measurements before and after taking the test samples. The key codes were strictly stored by the assignment manager (Y.N.) during the study, and the key was opened at the end of the study.

### 2.2. Participants

The participants were individuals who had subjective symptoms of xerostomia selected among the patients undergoing follow up visits at the Kagoshima University hospital between 7 July 2016 and 31 January 2019. Exclusion criteria were: (a) a history of radiation therapy or chemotherapy; (b) a history of food allergy, including pineapple; (c) a schedule of oral surgical operations, including extraction of teeth, during the research period; and (d) age below 20 years. Each participant received written and oral descriptions of the experimental protocols and provided written informed consent prior to enrollment.

We estimated the required sample size by using G* power 3.1.9.4 [17] assuming, on the basis of a previous study, that a medium effect size of 0.7 points for a mean difference in visual analog scale (VAS) score between with and without xerostomia symptoms could be detected [18]. The results revealed that *n* = 16 was the minimum sample size (alpha, 0.05; beta, 0.05 (95% power); two sides; dependent samples).

### 2.3. Test Sample

The test samples used in this study were tablets with GCP (GCP tablet) and without GCP (placebo tablet). Composition of the GCP tablet (500 mg) was: carbohydrate (0.2 g), and GCP (1.2 mg), for a total of 0.4 kcal. The composition of the placebo tablet (500 mg) was: carbohydrate (0.2 g), for a total of 0.4 kcal [12,13]. The GCP and placebo tablets were indistinguishable in terms of taste, flavor, and appearance including color, size, and packaging.

### 2.4. Assessment

The oral moisture level, xerostomia symptoms, and number of fungiform papillae were evaluated before (G1 and P1) and after (G2 and P2) taking the GCP and placebo test sample for two weeks, respectively. G1: Moisture level measured before taking GCP tablets, G2: Moisture level measured after taking GCP tablets, P1: Moisture level measured before taking Placebo tablets, P2: Moisture level measured after taking Placebo tablets. The evaluation methods are explained in detail below.

### 2.5. Oral Moisture Level

Measurements of oral moisture level were performed at the lingual mucosa by using an oral moisture-checking device (Mucus; Life Co., Ltd., Saitama, Japan). The measurements were performed 2 h after a meal, while the subject was in a stress-free environment [18,19]. The oral moisture level was measured three consecutive times, and the median was used as a representative value following a previous study [18].

### 2.6. Number of Fungiform Papillae

To assess the number of the fungiform papillae, a portable microscope with a 30× polarization/non-polarization lens (Scalar M3, Scalar, Tokyo, Japan) and a wooden tongue depressor (Star tongue depressor, Hoshiseido, Tokyo, Japan) were used. Participants were instructed to place their heads on the headrest of the dental chair, pull their tongue out to the extent that they did not feel pain, and hold their tongue firmly with their upper and lower lips. The assessor lightly placed the wooden tongue depressor on the participant’s tongue surface and drew the shape of the anterior margin of tongue on the tongue depressor surface. Based on a point at a distance of 10 mm from the center of the anterior margin of tongue drawn on the wooden tongue depressor, a square hole with sides of 4-mm length was made in the tongue depressor. The assessor placed the tongue depressor on the tongue again by using the drawn shape of the anterior margin of tongue as a reference, and took an image 4-mm length x 4-mm width with the microscope. The captured images were saved as JPEG files on a PC (Let’s Note SX2, Panasonic Corporation, Osaka, Japan), and the number of fungiform papillae with red capillary vessels was counted on these images [20]. The measurement was repeated three times, and the mean value was calculated.

### 2.7. Xerostomia Symptoms

Xerostomia symptoms, such as the degree of dryness in the mouth, the amount of saliva, the taste of the meal, and the satisfaction of oral moisture [1,2] were surveyed by using a 100-mm visual analog scale (VAS) by using the following questions: “How is the dryness of your mouth?”, “How is the secretion of saliva?”, “How was the taste of the meal?”, and “Are you satisfied with the moisture of your mouth?”. The worst condition was mapped at 0 mm, and the best condition was mapped at 100 mm on the VAS scale.

### 2.8. Statistical Analysis

Possible differences between the two groups at the baseline were assessed by using the t-test, the chi-square test, and the Mann–Whitney U-test. Possible carryover effects were analyzed by using the Wilcoxon signed-rank test for each of the measures taken in G1 and P1 in Sequence 1 and the measures taken in P1 and G1 in Sequence 2. The treatment effect was evaluated by analyzing each of the measures taken before and after taking GCP tablets and placebo tablets (i.e., G1 and G2 values, P1 and P2 values) by using the Wilcoxon signed-rank test. Statistical significance was set at α = 0.05. Data were analyzed by using SPSS v19 (IBM Japan Ltd., Tokyo, Japan).

## 3. Results

In total, 16 participants were randomized in the two groups and assessed from July 2016 to January 2019. Follow-up evaluations were completed in March 2019. No side effects caused by the intake of GCP tablets or placebo tablets were observed in any of the 16 participants. Table 1 outlines the baseline characteristics of the study participants in each group. There were no significant differences in age, number of remaining teeth, use of removable prostheses, medications, and oral moisture level at the baseline between the two groups, whereas the gender distribution was significantly different between the two groups.

Table 2 shows the results of the moisture level of the lingual mucosa. The upper part of the table shows the median, minimum, maximum, 25th and 75th percentile values, and 95% confidence interval (CI) of the moisture level at each evaluation point. The Wilcoxon signed-rank test revealed that there were no statistically significant differences in oral moisture levels measured in G1 and P1 in Sequence 1 and in P1 and G1 in Sequence 2, suggesting no carryover effects. The lower part of the table shows the median, minimum, maximum, 25th, and 75th percentile, and 95% CI of the moisture level before and after taking GCP and before and after taking placebo tablets. The Wilcoxon signed-rank test revealed that the moisture level measured after taking GCP tablets (in G2) was significantly higher than the one measured before (in G1), whereas no significant differences were observed after taking placebo tablets and (P2 versus P1).

Table 3 shows the results of the number of fungiform papillae. The upper part of the table shows the median, minimum, maximum, 25th and 75th percentile values, and 95% CI of the number of fungiform papillae at each evaluation point. The Wilcoxon signed-rank test revealed that there were no statistically significant difference in the number of fungiform papillae measured in G1 and P1 in Sequence 1 and in P1 and G1 in Sequence 2, suggesting no carryover effects. The lower part of the table shows the median, minimum, maximum, 25th and 75th percentile values, and 95% CI of the number of fungiform papillae measured before and after taking GCP tablets and placebo tablets. The Wilcoxon signed-rank test revealed that there were no statistically significant differences in the number of fungiform papillae measured before and after taking GCP tablets and placebo tablets.

Table 4 shows the results of the VAS values for a question “How is the dryness of your mouth?”. The upper part of the table shows the median, minimum, maximum, 25th and 75th percentile values, and 95% CI of the VAS values at each evaluation point. The Wilcoxon signed-rank test revealed that there were no statistically significant difference in the VAS values measured in G1 and P1 in Sequence 1 and in P1 and G1 in Sequence 2, suggesting no carryover effects. The lower part of the table shows the median, minimum, maximum, 25th and 75th percentile values, and 95% CI of the VAS values measured before and after taking GCP tablets and placebo tablets. The Wilcoxon signed-rank test revealed that the VAS values measured after taking GCP tablets (in G2) were significantly higher than those measured before (in G1), whereas no significant differences in VAS values were observed after taking placebo tablets (P2 versus P1). Similarly as in Table 4, Table 5, Table 6, and Table 7 show the results of the VAS values for the following questions: “How is the secretion of saliva?” (Table 5), “How was the taste of the meal?” (Table 6), and “Are you satisfied with the moisture of your mouth?” (Table 7). Similarly as in Table 4, here, the Wilcoxon signed-rank test revealed that there were no statistically significant differences in any of the VAS values measured in G1 and P1 in Sequence 1 and in P1 and G1 in Sequence 2, suggesting no carryover effects. In addition, the Wilcoxon signed-rank test revealed that there were no statistically significant differences among any of the VAS values measured before and after taking GCP tablets and placebo tablets.

## 4. Discussion

In this randomized cross-over test on patients with xerostomia symptoms, the effects of oral intake of GCP on oral moisture and xerostomia symptoms were compared with that of the ingestion of placebo tablets. As a result, the oral moisture significantly increased, and the visual analog scale (VAS) of “How is the dryness of your mouth?” significantly improved after GCP tablets intake and not after placebo tablets intake. The cross-over design used in this study has the advantages that the variation in data is smaller than that of a group comparison test, and the number of cases for obtaining results can be reduced. Based on previous studies [12,13], we set an eight-week study period (two-week intake and four-week washout) to ensure sufficient safety and an adequate washout period. It is widely known that carryover effects may occur in cross-over design studies. In these kinds of studies, it is important to assess whether the intervention effect is carried over, as it may occur even if a given washout period is set. Orally ingested glucosylceramide is absorbed in the intestine and taken up by epithelial cells [10]. Considering that the turnover rate of cells in the epithelial tissue of the tongue is about seven days [21], a safe washout period of four weeks was set in this study. In fact, results showed that there were no statistically significant differences in any of the measures here considered between the G1 and P1 values in Sequence 1 and the P1 and G1 values in Sequence 2, suggesting that the washout period here used was sufficient to avoid carryover effects.

This study has some limitations. Specifically, these include the relatively low number of subjects in each group (*n* = 8) and the relatively high oral moisture levels at the baseline. In this study, a high number of participants (35 individuals) were excluded, as they were not eligible based on the exclusion criteria. Specifically, these participants were postoperative patients with oral cancer who had a history of radiation therapy in the head and neck, which is a risk factor that causes salivary gland damage and xerostomia [1,2].

It is desirable that the examination of dry mouth be performed simply and quickly chair-side. Moisture-checking devices are available for such moisture measurements. Moisture-checking devices measure the moisture of the submucosal layer indirectly [18,19]. Oral moisture, which was recorded as the moisture value, was expressed as a percentage: B/(A + B) × 100(%), where A is the weight of the dried protein membrane, and B is the weight of the water [18]. It has significant advantages for the examination of oral moisture levels, because the measurement time is only 2 s, and is not affected by the patient’s chewing ability [18]. It is reported that the degree of moisture in the lingual mucosa is correlated with the salivary flow rates (unstimulated whole salivary flow and stimulated whole salivary flow) [19].

The participants in this study had an average age of about 70 years. Their main underlying disease was hypertension, and they were taking antihypertensive drugs. This is a risk factor for xerostomia [1,2]. However, there were no significant differences in the number of underlying disease and medications at the baseline between the two groups. Also, there was no significant differences in the moisture level at the baseline between the two groups. However, it is important to note that the median moisture level of these groups was 31.55, which is higher compared to the standard average value of xerostomia patients (27.9 or less) [19]. A study measured the moisture level of the lingual mucosa in patients with xerostomia symptoms by using an oral moisture-checking device, and showed that the moisture level was 27.0, i.e., lower than the values observed in this study [18]. However, those results were related to patients with jaw defects who received radiation therapy, and as such, they cannot be compared directly with those obtained in this study.

Moreover, according to the results of this study in which xerostomia symptoms were assessed by using a 100-mm VAS (i.e., the degree of dryness in the mouth, the amount of saliva, the taste of meal, and overall satisfaction of oral moisture), it also became clear that many participants were not satisfied with their oral environment. Some reported that xerostomia symptoms were correlated with the amount of stimulated and unstimulated saliva at rest, whereas others did not find any correlation; therefore, no consistent patterns were observed [18]. In this study, we set the participants’ xerostomia symptoms as inclusion criterion, because this criterion was easier to assess compared to the measurement of salivary secretion. The results of this study, in which a significant difference in gender distribution was found between the two groups at the baseline, might be attributed to the reasons described above. At the same time, these results are consistent with previous studies that have reported that women are more likely to experience xerostomia compared to men [1,2]. In order to clarify this point, gender stratification may be necessary when assigning subjects in future studies.

It is reported that an oral intake of glucosylceramide derived from konjac improves transepidermal water loss in health human subjects [9]. However, to date, no clinical studies have been conducted to examine the effects of oral ingestion of glucosylceramide on xerostomia. The GCP used in this study was a substance extracted and refined from pineapple fruit, i.e., a fruit that can be eaten daily. Regarding this, clearance has already been obtained for GCP as a food material [12,13]. In this study, tablets containing 1.2 mg of GCP, which have been tested for humans and have been confirmed for safety, were used [12,15]. Within the scope of our investigation, there have been no reports of health hazards due to GCP-containing foods, and no study using GCP has reported side effects. However, in general, there is a possible risk of allergic reactions to pineapple, and for this reason, patients with a history of allergies to fruits, including pineapple, were excluded. Overall, there were no reports of adverse events such as discomfort, feeling of strangeness, or other physical or mental abnormalities with the use of GCP tablets in this study.

The results of a randomized, double-blind, placebo-controlled, parallel-group comparison study of a 12-week oral intake of 1.2 mg of GCP in healthy Japanese adults who were concerned about skin dullness and dryness indicated decreases in transepidermal water loss, changes in color difference (increase in L* value, decrease in a* value in Lab color space), and increases in the number of textures on skin [15]. The reasons for the observed results were related to the skin structures becoming complete [15] due to increased ceramide synthesis [11] and the formation of cornified envelopes. In a similar way, it could be assumed that similar improvement effects could be produced on the lingual mucosa by the oral intake of GCP, as the lingual mucosa consists of the same stratum corneum squamous epithelium as the skin. The results of this study showed that GCP administration significantly increased the oral moisture level of the lingual mucosa and the VAS value related to xerostomia, but there was no significant change in the number of fungiform papillae after treatment. This may be due to the relatively high oral moisture level of the lingual mucosa at the baseline, as well as to differences in the GCP dosing period, but further investigations are necessary in this regard.

Xerostomia is caused by many factors, including in the elderly [1,2]. It has been shown that salivary gland function declines with age, even in the case of no systemic disease or medication [22]. Recently, in addition to the application of moisturizers [1,2,3], which is a well-established symptomatic treatment for the prevention of various symptoms [1,2] caused by xerostomia in the elderly, salivary gland massage and facial muscle and tongue exercises have also been reported as possible strategies to improve saliva secretion [23]. Each of these methods, as well as the GCP administration addressed in this study, have been studied in isolation in terms of their effects on xerostomia. In the future, it will be important to assess if, and to what extent, the beneficial effects of treatment for xerostomia may be enhanced by a combined application of more than one method. In addition, it is necessary to examine the effects of oral ingestion of glucosylceramide on the quality of life in patients with dry mouth symptoms.

## 5. Conclusions

Within the limitations of this study, the results suggested that the oral intake of glucosylceramide extracted from pineapple may improve the moisture level of the lingual mucosa and xerostomia symptoms.

## Figures and Tables

**Table 1:** Outline of study participants at baseline.

	Sequence 1 (GCP-Placebo)	Sequence 2 (Placebo-GCP)	Total	*p* Value
Patients; number	8	8	16	
Age; mean ± SD	73.3 ± 9.9	75.3 ± 4.6	74.3 ± 7.6	0.06 ^#^
Gender; Men/Women	1/7	4/4	5/11	0.00 ^$^
No. of remaining teeth (Upper)	6.3 ± 5.6	4.9 ± 4.3	5.6 ± 4.9	0.59 ^#^
No. of remaining teeth (Lower)	7.1 ± 4.8	6.7 ± 5.5	6.9 ± 5.0	0.89 ^#^
Use of removable prostheses: None/Upper or lower/Upper and lower	1/2/5	1/1/6	2/3/11	0.81 ^$^
Number of underlying diseases: None/One/More than two	2/ 3/ 3	1/6/1	3/9/4	0.31 ^$^
Number of medications: None/One/More than two	2/1/5	1/3/4	3/4/9	0.48 ^$^
Baseline oral moisture level: median (range)	G1: 31.55 (7.3)	P1: 31.55 (2.7)	31.55 (7.3)	0.48 ^%^

^#^ t-test, ^$^ chi-square test, ^%^ Mann–Whitney U-test. Statistical significance is assumed where *p* < 0.05.

**Table 2:** Moisture level of the lingual mucosa (%).

	Period	Median	Min	Max	25% Tile	75% Tile	95% CI	*p* Value
Sequence 1	G1	31.55	26.00	33.30	28.65	32.55	28.63–32.82	^#^*p* = 0.26 ^a^
(GCP-Placebo)	G2	32.20	29.00	33.10	31.33	32.98	30.75–33.02	
	P1	32.15	30.30	33.50	31.38	32.93	31.24–32.94	
	P2	32.60	30.50	33.90	31.38	33.70	31.47–33.56	
Sequence 2	P1	31.55	30.40	33.10	31.03	32.58	30.94–32.51	^#^*p* = 0.48 ^b^
(Placebo-GCP)	P2	31.80	31.20	33.40	31.25	33.05	31.35–32.85	
	G1	31.70	30.70	33.20	31.13	32.25	31.12–32.43	
	G2	32.40	28.80	33.40	31.20	33.18	30.68–33.24	
GCP	G1	31.70	26.00	33.30	30.78	32.38	30.23–32.25	^#^*p* = 0.03 ^c^
	G2	32.35	28.80	33.40	31.23	33.08	31.18–32.67	
Placebo	P1	31.90	30.30	33.50	31.35	32.60	31.46–32.41	^#^*p* = 0.12 ^d^
	P2	32.45	28.80	33.90	31.20	33.35	31.50–32.97	

^a^ G1 vs. P1, ^b^ P1 vs. G1: Carryover effect. ^c^ G1 vs. G2, ^d^ P1 vs. P2: Treatment effect. Moisture level of the lingual mucosa (%) at each evaluation point in Sequence 1 and Sequence 2. Upper part of the table: Moisture level of the lingual mucosa at each evaluation point in Sequence 1 and Sequence 2. Lower part of the table: Moisture level of the lingual mucosa before and after taking GCP tablets and placebo tablets. ^#^ Wilcoxon signed-rank test. Statistical significance is assumed where *p* < 0.05.

**Table 3:** Number of fungiform papillae.

	Period	Median	Min	Max	25% Tile	75% Tile	95% CI	*p* Value
Sequence 1	G1	8.5	6.0	14.0	7.3	10.5	6.9–11.1	^#^*p* = 0.32 ^a^
(GCP-Placebo)	G2	8.0	6.0	14.0	7.3	10.5	6.8–11.0	
	P1	8.0	6.0	15.0	6.5	9.8	6.4–11.1	
	P2	8.0	6.0	15.0	7.3	9.8	6.6–11.2	
Sequence 2	P1	12.5	5.0	25.0	8.3	14.5	7.5–17.5	^#^*p* = 0.32 ^b^
(Placebo-GCP)	P2	11.5	6.0	25.0	8.3	14.3	7.4–17.1	
	G1	12.0	5.0	25.0	8.3	14.5	7.4–17.4	
	G2	12.5	4.0	26.0	8.3	14.8	7.2–18.1	
GCP	G1	9.0	5.0	25.0	8.0	12.8	8.1–13.2	^#^*p* = 0.66 ^c^
	G2	9.0	4.0	26.0	8.0	13.8	8.0–13.5	
Placebo	P1	9.0	5.0	25.0	8.0	13.0	8.0–13.3	^#^*p* = 0.71 ^d^
	P2	9.0	6.0	25.0	8.0	12.0	8.0–13.1	

^a^ G1 vs. P1, ^b^ P1 vs. G1: Carryover effect. ^c^ G1 vs. G2, ^d^ P1 vs. P2: Treatment effect. Upper part of the table: The number of fungiform papillae at each evaluation point in Sequence 1 and Sequence 2. Lower part of the table: The number of fungiform papillae before and after taking GCP tablets and placebo tablets. ^#^ Wilcoxon signed-rank test. Statistical significance is assumed where *p* < 0.05.

**Table 4:** VAS values for a question “How is the dryness of your mouth?”

	Period	Median	Min	Max	25% Tile	75% Tile	95% CI	*p* Value
Sequence 1	G1	42.86	9.54	78.99	12.55	61.54	18.82–62.03	^#^*p* = 0.09 ^a^
(GCP-Placebo)	G2	43.05	22.20	73.18	24.45	70.68	27.42–65.91	
	P1	54.67	3.43	87.57	46.04	71.13	33.38–74.64	
	P2	60.31	8.44	86.99	24.05	74.96	27.75–74.64	
Sequence 2	P1	52.99	8.48	88.95	35.06	62.29	30.57–69.92	^#^*p* = 1.00 ^b^
(Placebo-GCP)	P2	44.71	33.51	94.62	36.57	59.60	33.99–67.43	
	G1	50.45	19.28	90.94	29.89	62.96	30.56–69.41	
	G2	49.81	34.38	92.26	37.97	68.66	37.46–70.50	
GCP	G1	46.02	9.54	90.94	22.23	62.91	32.29–58.13	^#^*p* = 0.04 ^c^
	G2	49.81	22.20	92.26	30.23	70.68	39.10–61.55	
Placebo	P1	54.05	3.43	88.95	44.63	63.42	39.67–64.58	^#^*p* = 0.96 ^d^
	P2	48.19	8.44	94.62	33.97	64.47	38.43–63.58	

^a^ G1 vs. P1, ^b^ P1 vs. G1: Carryover effect. ^c^ G1 vs. G2, ^d^ P1 vs. P2: Treatment effect. Upper part of the table: VAS values at each evaluation point in Sequence 1 and Sequence 2. Lower part of the table: VAS values before and after taking GCP tablets and placebo tablets. ^#^ Wilcoxon signed-rank test. Statistical significance is assumed where *p* < 0.05.

**Table 5:** VAS values for the question, “How is your secretion of saliva?”.

	Period	Median	Min	Max	25% Tile	75% Tile	95% CI	*p* Value
Sequence 1	G1	37.97	9.16	79.40	10.03	62.73	15.37–60.95	^#^*p* = 0.12 ^a^
(GCP-Placebo)	G2	63.63	14.40	80.00	43.05	72.54	39.32–75.77	
	P1	55.34	3.44	86.77	46.84	68.58	33.33–73.72	
	P2	61.97	8.80	87.94	47.56	78.04	38.96–80.30	
Sequence 2	P1	51.19	10.68	89.66	20.60	66.10	25.48–69.98	^#^*p* = 0.48 ^b^
(Placebo-GCP)	P2	46.14	30.96	95.41	39.03	68.41	35.29–70.83	
	G1	52.33	21.71	91.36	37.94	70.28	36.11–72.66	
	G2	45.93	39.01	90.97	41.15	76.54	38.11–72.55	
GCP	G1	46.73	9.16	91.36	24.51	64.62	32.79–59.75	^#^*p* = 0.09 ^c^
	G2	54.62	14.40	90.97	41.15	72.54	45.50–67.37	
Placebo	P1	55.34	3.44	89.66	38.91	67.26	37.45–63.81	^#^*p* = 0.31 ^d^
	P2	54.69	8.80	95.41	42.44	75.50	44.34–68.35	

^a^ G1 vs. P1, ^b^ P1 vs. G1: Carryover effect. ^c^ G1 vs. G2, ^d^ P1 vs. P2: Treatment effect. Upper part of the table: VAS values at each evaluation point in Sequence 1 and Sequence 2. Lower part of the table: VAS values before and after taking GCP tablets and placebo tablets. ^#^ Wilcoxon signed-rank test. Statistical significance is assumed where *p* < 0.05.

**Table 6:** VAS values for the question, “How was the taste of the meal?”.

	Period	Median	Min	Max	25% Tile	75% Tile	95% CI	*p* Value
Sequence 1	G1	74.62	45.05	98.27	50.45	91.61	55.72–90.01	^#^*p* = 0.09 ^a^
(GCP-Placebo)	G2	66.08	51.66	98.71	52.94	78.78	54.38–81.56	
	P1	63.28	20.49	95.98	53.59	74.11	44.50–80.76	
	P2	71.35	26.49	97.64	63.37	89.93	52.92–90.47	
Sequence 2	P1	78.54	47.69	98.63	74.21	89.26	66.04–91.13	^#^*p* = 0.58 ^b^
(Placebo-GCP)	P2	75.77	50.35	94.81	53.11	93.12	57.10–89.50	
	G1	78.03	49.22	97.34	56.46	87.93	60.47–88.97	
	G2	72.56	43.54	91.17	51.68	89.57	56.00–87.43	
GCP	G1	76.73	45.05	98.27	53.17	91.13	64.07–83.52	^#^*p* = 0.31 ^c^
	G2	70.57	43.54	98.71	52.94	86.33	60.74–78.95	
Placebo	P1	74.05	20.49	98.63	58.93	83.43	60.05–81.16	^#^*p* = 0.68 ^d^
	P2	72.45	26.49	97.64	55.72	90.07	61.69–83.30	

^a^ G1 vs. P1, ^b^ P1 vs. G1: Carryover effect. ^c^ G1 vs. G2, ^d^ P1 vs. P2: Treatment effect. Upper part of the table: VAS values at each evaluation point in Sequence 1 and Sequence 2. Lower part of the table: VAS values before and after taking GCP tablets and placebo tablets. ^#^ Wilcoxon signed-rank test. Statistical significance is assumed where *p* < 0.05.

**Table 7:** VAS values for the question, “Are you satisfied with the moisture level of your mouth?”.

	Period	Median	Min	Max	25% Tile	75% Tile	95% CI	*p* Value
Sequence 1	G1	48.82	7.25	90.48	15.64	70.66	22.12–72.36	^#^*p* = 0.41 ^a^
(GCP-Placebo)	G2	65.94	20.84	89.72	27.59	81.25	35.16–80.94	
	P1	60.41	3.16	85.98	34.41	78.92	32.41–79.03	
	P2	60.28	8.20	91.11	41.97	74.87	35.74–77.99	
Sequence 2	P1	40.39	10.05	91.56	18.31	85.61	20.44–76.84	^#^*p* = 0.48 ^b^
(Placebo-GCP)	P2	45.82	30.95	93.98	35.70	86.51	35.72–79.11	
	G1	51.98	25.97	89.05	29.51	77.09	33.47–73.70	
	G2	52.13	27.40	90.07	38.61	80.93	37.17–75.08	
GCP	G1	51.98	7.25	90.48	29.21	70.66	36.29–64.53	^#^*p* = 0.35 ^c^
	G2	55.47	20.84	90.07	36.60	81.25	44.14–70.04	
Placebo	P1	56.44	3.16	91.56	24.02	80.23	36.13–68.23	^#^*p* = 0.23 ^d^
	P2	55.20	8.20	93.98	38.13	80.66	43.95–70.33	

^a^ G1 vs. P1, ^b^ P1 vs. G1: Carryover effect. ^c^ G1 vs. G2, ^d^ P1 vs. P2: Treatment effect. Upper part of the table: VAS values at each evaluation point in Sequence 1 and Sequence 2. Lower part of the table: VAS values before and after taking GCP tablets and placebo tablets. ^#^ Wilcoxon signed-rank test. Statistical significance is assumed where *p* < 0.05.
